# MAPT mutations associated with familial tauopathies lead to formation of conformationally distinct oligomers that have cross‐seeding ability

**DOI:** 10.1002/pro.5099

**Published:** 2024-08-15

**Authors:** Anukool A. Bhopatkar, Nemil Bhatt, Md Anzarul Haque, Rhea Xavier, Leiana Fung, Cynthia Jerez, Rakez Kayed

**Affiliations:** ^1^ Department of Neurology, Mitchell Center for Neurodegenerative Diseases University of Texas Medical Branch Galveston Texas USA; ^2^ Departments of Neurology, Neuroscience and Cell Biology University of Texas Medical Branch Galveston Texas USA; ^3^ Present address: Department of Pharmacology and Toxicology University of Mississippi Medical Center Jackson Mississippi USA; ^4^ Present address: Neuroscience Graduate Program, UT Southwestern Medical Center Dallas Texas USA

**Keywords:** conformation, cross‐seeding, frontotemporal dementia, MAPT, oligomers, tauopathy

## Abstract

The microtubule associated protein, tau, is implicated in a multitude of neurodegenerative disorders that are collectively termed as tauopathies. These disorders are characterized by the presence of tau aggregates within the brain of afflicted individuals. Mutations within the *MAPT* gene that encodes the tau protein form the genetic backdrop for familial forms of tauopathies, such as frontotemporal dementia (FTD), but the molecular consequences of such alterations and their pathological effects are unclear. We sought to investigate the conformational properties of the aggregates of three tau mutants: A152T, P301L, and R406W, all implicated within FTD, and compare them to those of the native form (WT‐Tau 2N4R). Our immunochemical analysis reveals that mutants and WT tau oligomers exhibit similar affinity for conformation‐specific antibodies but have distinct morphology and secondary structure. Additionally, these oligomers possess different dye‐binding properties and varying sensitivity to proteolytic processing. These results point to conformational variety among them. We then tested the ability of the mutant oligomers to cross‐seed the aggregation of WT tau monomer. Using similar array of experiments, we found that cross‐seeding with mutant aggregates leads to the formation of conformationally unique WT oligomers. The results discussed in this paper provide a novel perspective on the structural properties of oligomeric forms of WT tau 2N4R and its mutant, along with shedding some light on their cross‐seeding behavior.

## 1 INTRODUCTION

Tauopathies represent a group of neurodegenerative disorders typified by the aggregated deposits of the microtubule associated protein, tau, within the brain of afflicted individuals (Kovacs, 2017; V.M.Y. Lee et al., 2001; Zhang et al., 2022). Tau protein is normally bound to microtubules and helps stabilize their assembly (Guo et al., 2017; V.M.Y. Lee et al., 2001; Trojanowski and Lee, 2005). In pathology, misfolding of the protein and aggregation leads to the loss of this function (Guo et al., 2017; Luo et al., 2016; Trojanowski and Lee, 2005). Humans express six isoforms of tau which differ from each other by their lengths; three isoforms with three microtubule binding repeats, termed as 3R, and three isoforms with four repeats, termed as 4R. These isoforms serve as the basis of categorizing tauopathies; depending on which tau isoform is predominant in the pathogenic deposits, they are categorized as 3R‐tauopathies, 4R‐tauopathies, or mixed tauopathies (Zhang et al., 2022). Tauopathies are also classified as primary tauopathies if tau is the predominant species in aggregates, such as frontotemporal dementia (FTD), Pick's disease, corticobasal degeneration, chronic traumatic encephalopathy among others. In secondary tauopathies, such as Alzheimer's disease (AD), the aggregation and deposition of tau is the secondary pathological alteration (aggregation of amyloid‐beta [Αβ] is considered the primary insult in this case).

Multiple mutant forms of tau have been identified in familial tauopathies (Poorkaj et al., 2001; Wolfe, 2009). Among them, studies have reported that two of these mutants, P301L (also the most common mutation observed in the familial form [Poorkaj et al., 2001]), and R406W, were found to co‐deposit with native tau in the brain of patients affected with FTD (Miyasaka et al., 2001a, 2001b). This observation potentially suggests WT and mutant forms of tau might have a reciprocal influence on the aggregation of the other. Such an occurrence is not novel for tau, since studies have identified that multiple tau isoforms are present within tau tangles (Goedert et al., 1989), and a recent report by Dregni et al. (2022) found that 3R and 4R isoforms of the protein can form mixed aggregates while adopting a common conformation. Despite this, the heterogeneity in aggregate structure is evident in tauopathies (Shi et al., 2021). This structural variety in the amyloid form of individual proteins, termed as polymorphism, has only recently gained relevance within the field of neurodegenerative disorders (Close et al., 2018; Tycko, 2015). As such, they are becoming the focus of investigations as potential therapeutic targets to combat these fatal disorders. However, progress is hampered by the scarcity of information on the multitude of conformational ensembles that are possible for each isoform, and the various factors that engender polymorphism. Further complexity is added by the fact that there is now a consensus within the field that oligomers, and not fibrils, are the most potent pathological forms (Bhatt et al., 2024; Caughey and Lansbury, 2003; Ghag et al., 2018; Kayed et al., 2004; Kirkitadze et al., 2002; Klein et al., 2004; Xue et al., 2009). The thorough characterization of these ephemeral, soluble aggregates represent a biophysical challenge, but progress has been made on that front by many groups (Bhatt et al., 2024; Glabe, 2008; Kayed et al., 2003, 2009; Laganowsky et al., 2012). Recent studies have shown that multiple amyloid proteins can interact with each other and affect the aggregation kinetics and structural aspects of the final amyloid form that emerges; Aβ interacts with α‐synuclein (αS), tau, and other proteins (Luo et al., 2016); tau and αS have been shown to synergistically alter the aggregation of each other (Giasson et al., 2003; Lu et al., 2020), and interaction of Aβ and prion protein have reciprocal effects on their aggregate formation (Chen et al., 2010). Also, Castillo‐Carranza et al. (2018) shed light on how αS induces polymorphism in tau oligomers, which have unique cytotoxicity. Other studies have also shown how small molecules can modulate the aggregation and conformation of tau oligomers (Lo Cascio et al., 2019, 2020).

Being cognizant of these findings, we endeavored to investigate the conformational properties of the aggregates formed by three mutant forms of tau 2N4R (the longest isoform): (a) tau A152T, which was recently identified as a risk factor for FTD and AD (Coppola et al., 2012), (b) tau P301L, and (c) tau R406W, along with the native form. Additionally, we tested if the oligomers of the mutant proteins can influence the aggregation of WT tau. To do this, we expressed and purified recombinant tau and its mutant forms followed by generating their soluble aggregates (oligomers). We then utilized biophysical and biochemical tools to characterize their conformational properties. Our results reveal that conformational differences exist among the oligomers, although they have similar immunochemical properties based on western‐blot and dot blot analysis. Interestingly, using mutant tau oligomers as seeds in the aggregation of WT tau resulted in formation of oligomers of the latter that are different from the unseeded ones. As before, we see similar immunochemical reactivity, but distinct morphology, secondary structure, dye‐binding ability, and proteolytic stability. These studies represent an important first step in characterizing the interactions between mutant and WT forms of tau, in addition to highlighting conformational variety of their oligomers.

## 2 RESULTS

### 2.1 Oligomers of WT tau and mutants show similar immunochemical affinities

**FIGURE 1:**
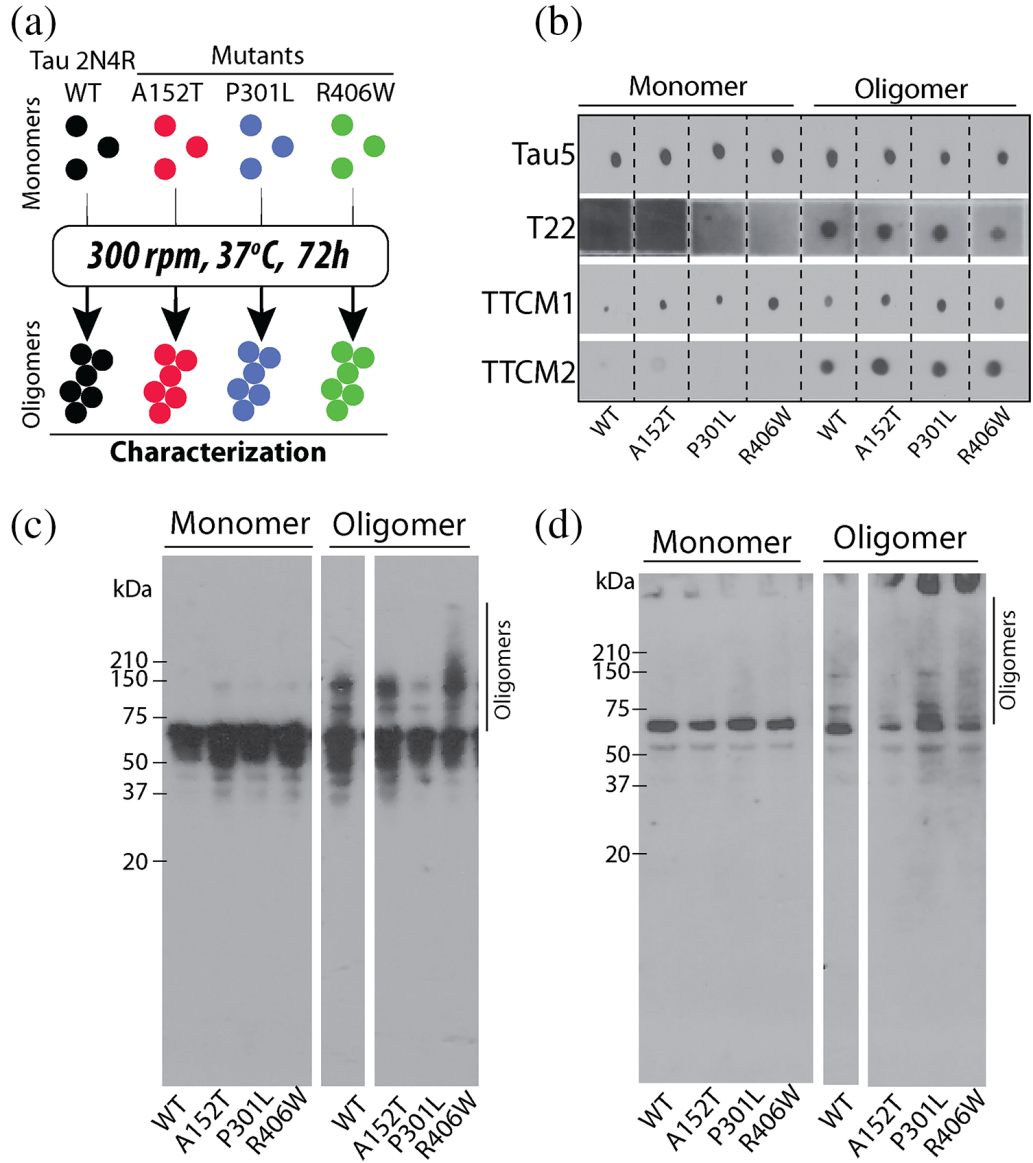
In‐vitro generation and characterization of WT‐Tau 2N4R and mutant oligomers. (a) Oligomers of WT‐Tau 2N4R and mutants (A152T, P301L, and R406W) were generated using recombinant monomers which were rotated at 300 rpm, 37°C for 72 h. The oligomers were then characterized via various biochemical, biophysical, and biological assays. (b) Dot‐blot assay for monomers and oligomers of WT tau and tau mutants performed using total tau (Tau5) and conformation specific anti‐oligomer antibodies (T22, TTCM‐1, TTCM‐2). (c and d) Western blot analyses for monomeric and oligomeric forms of WT and tau mutants probed using Tau5 (c) and T22 (d) antibodies. Oligomeric species are indicated with a solid vertical line.

To obtain the oligomers of WT tau 2N4R and its mutants, A152T, P301L, and R406W, we recombinantly expressed the monomeric forms of these proteins in bacteria (*Escherichia coli*). Next, using affinity chromatography, we isolated monomers of the protein and determined their purity by sodium dodecyl sulfate‐polyacrylamide gel electrophoresis (SDS‐PAGE) followed by silver staining (Figure S1 in Data S1). We then induced aggregation of the pure, unaggregated monomers by shaking them at 37°C for 72 h followed by characterization of the oligomers formed (Figure 1a–d). Firstly, we utilized a spectrum of antibodies to probe the immunochemical properties of the oligomers using a dot‐blot assay (Figure 1b). Along with a total tau antibody (Tau5) which is sequence specific, we used conformation‐specific antibodies directed against oligomeric forms of tau: T22, TTCM‐1, and TTCM‐2. For this, 4 μg of each protein was applied onto a nitrocellulose membrane and detected using the antibodies described. The results show that Tau5 has a uniform affinity for the monomeric and oligomeric form of WT tau 2N4R and its mutant, as would be expected. The conformation specific antibody, T22, detected only the oligomeric forms of WT and mutant tau, but failed to show any notable differences in signal intensity among them (Figure 1b). We observed similar immunoreactivity of TTCM‐1 with the monomeric and oligomeric forms of WT tau and its mutants, while TTCM‐2 specifically recognized all oligomers (Figure 1b). Additionally, we also performed western blot analysis to discern the size distribution of aggregates within our samples (Figure 1c,d). The western blot probed with Tau5 showed an intense band around ~50–75 kDa, more prominently seen in monomeric samples as compared to the oligomeric ones (Figure 1c). Samples from aggregation reactions showed higher molecular weight species prominent at 150 kDa, while larger aggregates were also visible (Figure 1c). This banding pattern is absent from monomeric samples, suggesting that these species represent aggregated products. Utilizing the conformation specific T22 antibody, we detected misfolded monomeric species visible as a single band at ~65–70 kDa (Figure 1d) while in our aggregated samples, we observed the presence of higher molecular weight species, appearing at 150 kDa (Figure 1d). Comparing the results of Tau5 and T22, we observe weaker immunoreactivity of the oligomeric forms for the latter antibody compared to the former. Overall, the results indicate that the oligomers of WT tau and its mutant share similar immunochemical properties.

### 2.2 Oligomers of WT tau and its mutants have distinct morphology and secondary structure

**FIGURE 2:**
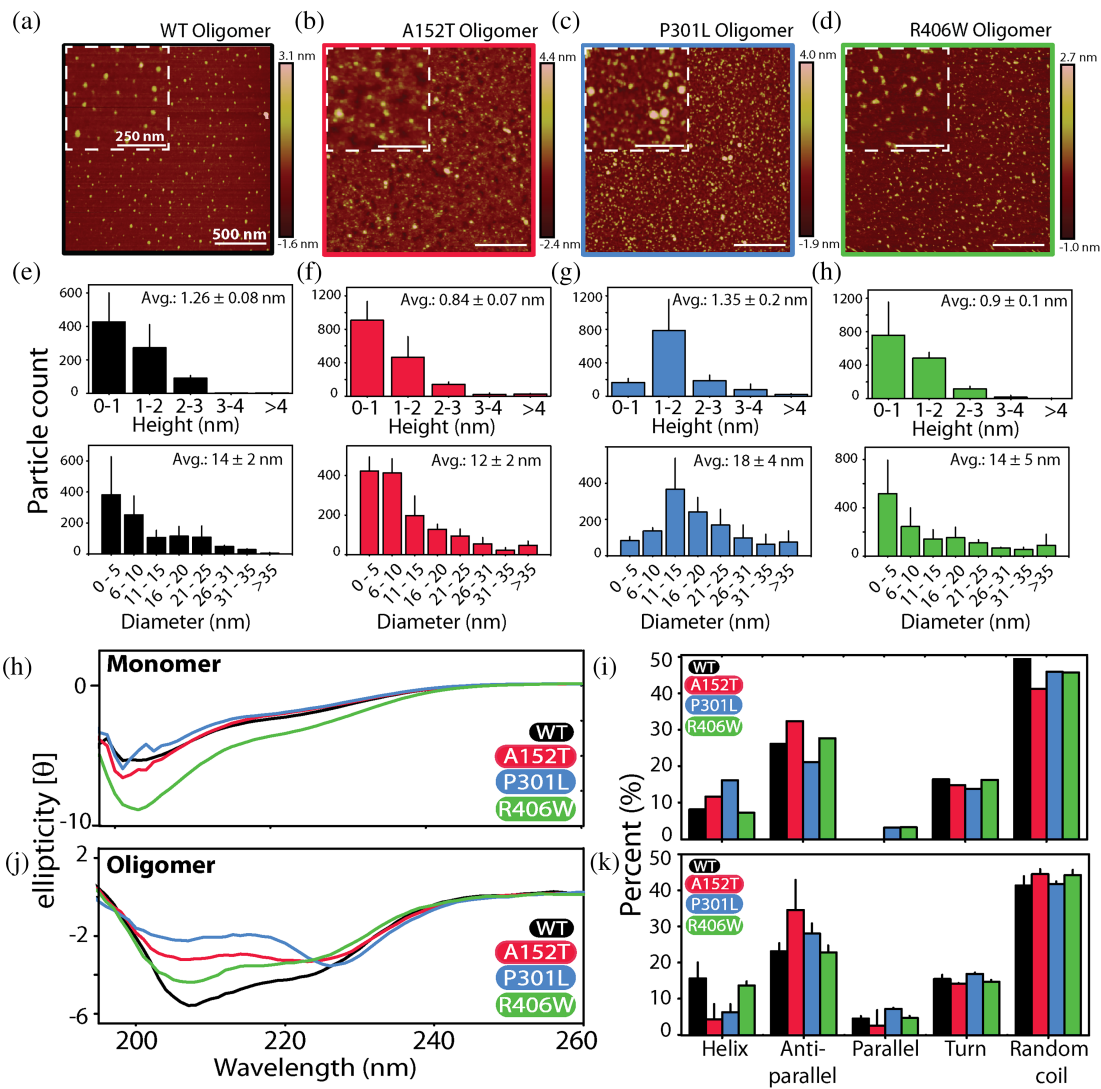
Tau mutant oligomers show distinct morphology and secondary structure. (a–d) Atomic force microscopy analysis of oligomeric samples of WT tau (a, black), A152T (b, red), P301L (c, blue), and R406W (d, green). Scale bars represent 500 nm. Insets show magnified area of the field where the scale bars represent 250 nm. (e–h) Morphological analysis (height and diameter) performed on micrographs of individual samples using the NanoScope v1.20rl software. (h–k) Far‐UV CD spectra of monomers (h) and oligomers (j) along with their deconvolution to identify secondary structural elements within each sample. Deconvolution analysis was performed using the online platform BeStSel. Error bars represent a mean of triplicate readings. CD, circular dichroism.

Further, we interrogated the morphology of the oligomers of WT tau and its mutants using atomic force microscopy (AFM) (Figure 2a–d). The representative micrographs of individual oligomeric samples are displayed in Figure 2a–d, where visual inspection reveals unique particle morphologies. We then subjected three to four micrographs (2 × 2 μm) of each sample to detailed particle analysis using NanoScope v1.20rl to tease out differences in their dimensions (Figure 2e–h). The results show that WT tau oligomers have a mean height of 1.26 nm (±0.1 nm) and mean diameter of 14 nm (±2 nm) (Figure 2e). The oligomers of tau A152T have mean height of 0.8 nm (±0.07 nm) and a mean diameter of 12 nm (±2 nm) (Figure 2f). The analysis revealed that the oligomeric particles of tau P301L have the largest dimensions; a mean height of 1.35 nm (±0.2 nm) and a mean diameter of 18 nm (±4 nm) (Figure 2g). Finally, the oligomers of tau R406W were found to have a mean height of 0.9 nm (±0.1 nm) and a mean diameter of 14 nm (±5 nm) (Figure 2h).The particle analysis confirms that individual oligomeric samples have differences in their overall dimensions.

We also examined the secondary structure of these oligomers using far‐UV (190–260 nm) circular dichroism (CD) (Figure 2h–j). The spectra of monomeric forms of all proteins show a prominent minimum at 200 nm, indicative of random coil (Figure 2h). We quantified the contribution of different secondary structural elements to the spectrum using the BeStSel platform (Micsonai et al., 2015). The analysis shows that random coil‐like features make up approximately half (~50%) of the total structure in the monomers (Figure 2h). Anti‐parallel β‐sheet constitutes between 20% and 30% of the structure, with exceedingly small amounts of parallel β‐sheet content, as would be expected (Figure 2i). Next, α‐helical segments made up between 5% and 15%, while turns and bends account for 10%–20% of the peptides (Figure 2i). The CD spectra of oligomeric samples show deviation from monomers, with stark differences among the oligomers themselves (Figure 2j). As seen in Figure 2j, the prominent minima observed differed from ~225 nm (for P301L, blue) to ~208 nm (for WT tau, black). Deconvolution of the spectra reveals a slight decrease in random coil content in oligomers and an increase in the amount of parallel β‐sheet structure (Figure 2k). The helical content of WT tau and R406W is higher in their oligomers compared to monomers, while the opposite is true for tau A152T and tau P301L. The results show that anti‐parallel β‐sheet represents the predominant structural feature of WT and mutant tau oligomers.

### 2.3 WT tau and mutant oligomers display differences in their dye‐binding ability and proteolytic stability

**FIGURE 3:**
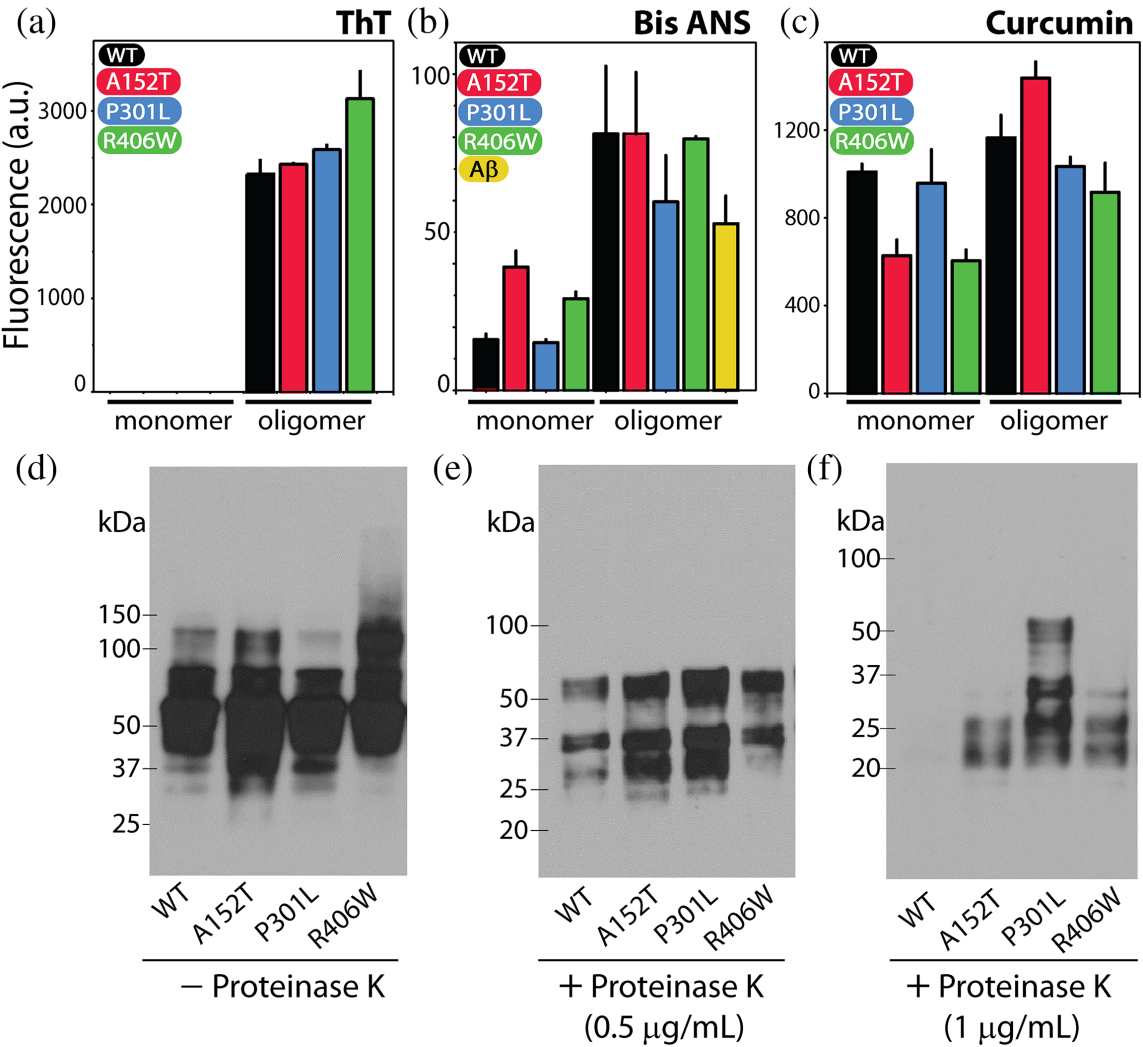
Oligomers of WT tau and its mutants display unique conformational signatures. (a–c) Fluorescence intensities of the dyes; ThT (a), bis‐ANS (b), and curcumin (c) in presence of monomers and oligomer of WT tau and mutants. Amyloid‐beta (Aβ) oligomers were used as positive control in the bis‐ANS assay (yellow bar, b). (d–f) Proteolytic stability of oligomers was tested by incubating them at 37°C for 30 min in presence of increasing concentrations of the enzyme proteinase K (PK): no PK (d), 0.5 μg/mL PK (e), and 1 μg/mL PK (f). The blot was probed with Tau5 antibody. Error bars represent a mean of triplicate readings. The western blot analysis presented here is consistent with three repeats.

Buoyed by the results of our morphological and structural analyses (Figure 2), we undertook a more detailed study of the oligomers by specifically focusing on their conformational properties (Figure 3). First, we used thioflavin‐T (ThT), a dye that fluoresces strongly at 482 nm (excitation max = 450 nm) upon binding to the cross‐β‐sheet structure (Barton et al., 2019; Biancalana and Koide, 2010) (Figure 3a). This assay allowed us to ascertain the amyloid content within individual oligomers and thus validate our far‐UV CD results (Figure 2j,k). The results show that none of our monomeric samples had any detectable ThT fluorescence, in turn indicating an absence of amyloid‐structures (Figure 3a). On the other hand, we see discernible fluorescence signals in oligomer samples which display similar, but not the same, ThT signal intensities (Figure 3a). In the presence of oligomers of R406W, ThT displayed the highest fluorescence intensity with a value of 3129 ± 306 a.u., while those in the presence of the rest of the oligomers were within the range of 2300–2500 a.u. (WT tau, 2323 ± 163 a.u.; A152T, 2432 ± 21 a.u.; P301L, 2586 ± 62 a.u.). We also used amyloid‐beta 42 fibrils (Aβ42f) as a positive control due to the well‐known biophysical characteristics (Figure S4A in Data S1). The Aβ42f sample showed a ThT intensity of 99,920 ± 10,922 a.u., which was ~30–40 fold that of the oligomers tested (Figure S4A in Data S1), affirming the presence of intermediate aggregation species within our samples.

We also examine the conformational properties of individual oligomers using 4,4′‐dianilino‐1,1′‐binaphthyl‐5,5′‐disulfonic acid (bis‐ANS), which recognizes exposed hydrophobic pockets on proteins (Bothra et al., 1998) (Figure 3b). In a nonpolar environment, the emission intensity of bis‐ANS is significantly elevated (Bothra et al., 1998). In the presence of the monomeric forms of the protein, bis‐ANS had varying intensities; with WT tau monomer sample, it was 16 ± 2 a.u., and with A152T monomer sample it was 39 ± 5a.u (Figure 3b). In the presence of P301L monomer sample, bis‐ANS had an intensity of 15 ± 11 a.u., while in presence of R406W monomer, the fluorescence intensity was 29 ± 2 a.u (Figure 3b). In presence of oligomers, we observed WT tau sample had the highest bis‐ANS emission intensity of 81 ± 21 a.u., which was very similar to that observed in presence of A152T oligomer (81 ± 20 a.u.) (Figure 3b). In the presence of P301L oligomers, bis‐ANS had a fluorescence intensity of 56 ± 15 a.u., while that in the presence of R406W oligomers was 79 ± 8 a.u (Figure 3b). Comparing the fluorescence intensity of these oligomer samples to that in the presence of Aβ42f, we see the latter sample had a lower bis‐ANS intensity (53 ± 9 a.u.) (Figure 3b).

Furthermore, we studied the emission intensity of the curcumin dye in presence of monomeric and oligomeric samples of WT tau and its mutants (Figure 3c). This dye has been used in the field of amyloid research to study conformational heterogeneity in amyloid species based on its differential emission between 500 and 550 nm when excited at 467 nm (Condello et al., 2018; Saha et al., 2021). In the presence of WT tau monomer, we observed curcumin emission intensity of 1007 ± 37 a.u., while in the presence of A152T monomer it was 627 ± 72 a.u. (Figure 3c). We saw an emission signal of 657 ± 152 a.u. in presence of P301L monomer and that in presence of R406W monomer, it was 604 ± 50 a.u. (Figure 3c). Importantly, we observed differences in fluorescence emission of curcumin in the presence of oligomeric samples we tested (Figure 3c). In presence of WT tau oligomer, we saw a fluorescence signal of 1164 ± 103 a.u., while in the presence of A152T oligomers, it was 1436 ± 74 a.u., which was the highest value among the oligomers (Figure 3c). In the sample containing P301L oligomers, we saw a fluorescence value of 1034 ± 42 a.u., and finally, the emission signal was 916 ± 133 a.u. in the presence of R406W oligomers, which was the lowest value observed (Figure 3c). Overall, the results from these assays hint at subtle differences in conformation adopted by individual oligomers.

Another tool to study the conformational properties of amyloid species is the measure of their proteolytic stability. We performed this assay by incubating the oligomers with increasing concentrations of the proteolytic enzyme, proteinase K (PK) (Figure 3d–f). We then performed western‐blot analysis on the samples using Tau5 (total tau antibody). As seen in Figure 3d, without PK, the oligomers of tau and its mutant are intact, with a smeared banding pattern extending from 50 to 150 kDa and above. No bands are prominently visible below the 37 kDa mark (Figure 3d). In presence of 0.5 μg/mL of PK, we see digestion of oligomers with a complete absence of bands above ~75 kDa (Figure 3e). Differences in the banding pattern for individual oligomers are not prominent here, but one can glean a difference in the band at ~30 kDa among the samples (Figure 3e). This band is prominently seen in oligomer samples of A152T and P301L, while it is less so in samples of WT tau and R406W oligomers (Figure 3e). The unique proteolytic stability of each oligomer is evident in the presence of 1 μg/mL PK (Figure 3f). Here, we see the oligomer of WT tau is completely digested, while faint bands are observed between 20 and 25 kDa mark for A152T oligomer (Figure 3f). The oligomers of P301L show starkly different stability in the presence of 1 μg/mL PK, with proteolysis resistant bands observed at ~50, ~30 and ~25 kDa (Figure 3f). Finally, in the R406W sample, faint bands of proteolysis resistant species are visible at ~30 kDa, and between 20 and 25 kDa (Figure 3f). The unique proteolytic signature of individual oligomers highlights the conformational heterogeneity among them.

### 2.4 Cross‐seeded WT tau oligomers are immunochemically similar to unseeded form

**FIGURE 4:**
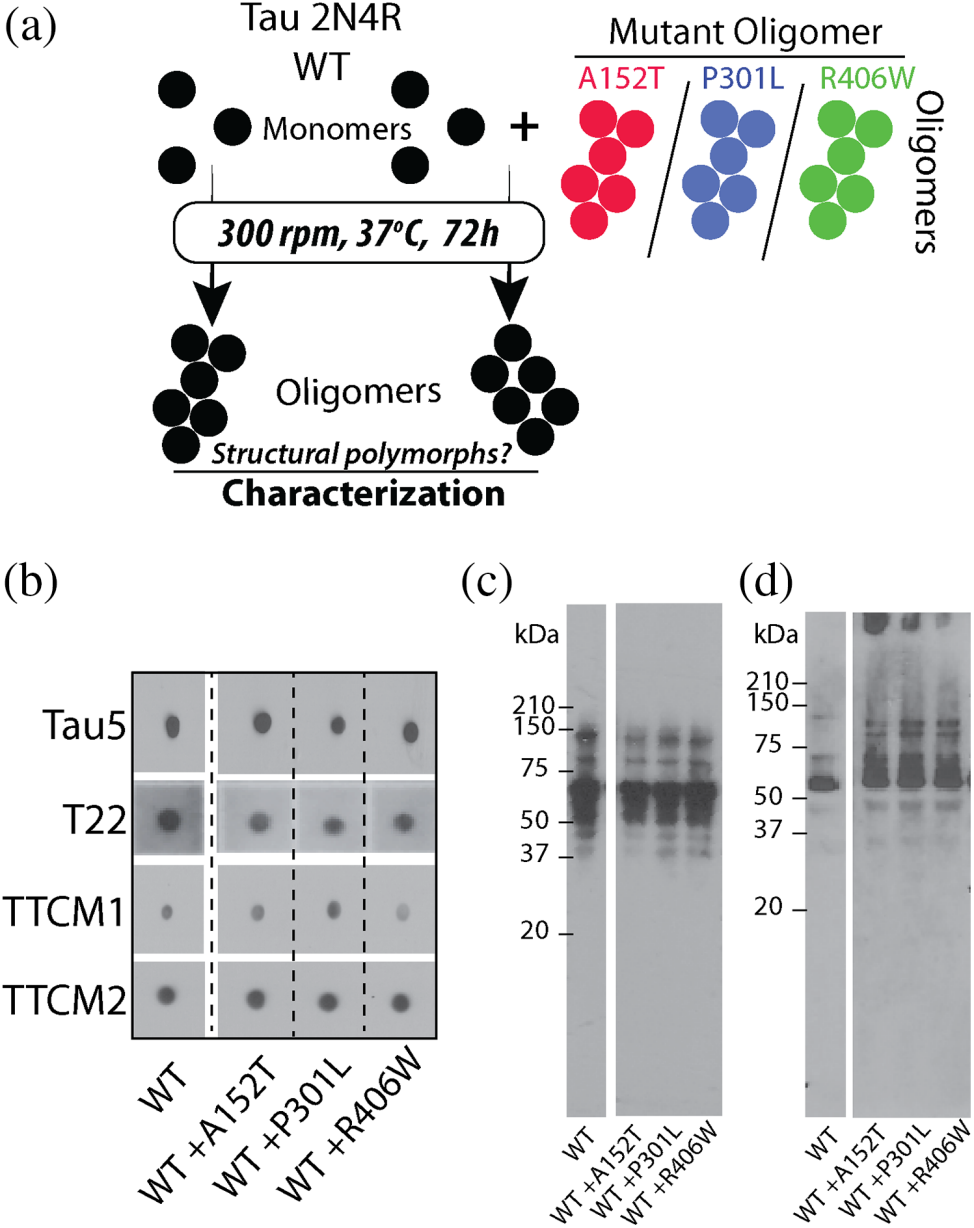
Generation and characterization of cross‐seeded WT tau oligomers. (a) Monomeric WT tau was incubated with tau mutant oligomers (100/1 monomer/oligomer ratio) at 37°C for 72 h with continuous shaking at 300 rpm to generate cross‐seeded oligomers which were further characterized. (b–d) Immunochemical characterization of WT tau oligomers generated in presence of tau mutant oligomers using dot‐blot assay (b) and western‐blot analysis with Tau5 (c) and T22 (d) antibodies. The dot‐blot and western‐blot data for the WT tau alone have been reproduced from Figure 1. Refer Figure S3 in Data S1 for the complete blots.

Reports have identified the co‐localized presence of native tau protein with its mutant isoforms in aggregates within the brain of patients afflicted with tauopathies (Miyasaka et al., 2001a, 2001b). Here we investigated the possibility that aggregated forms of tau mutants can potentially seed the aggregation of WT tau. To generate such cross‐seeded species, we incubated the oligomers of mutant tau with WT tau monomer (1:100 oligomer:monomer) at 37°C for 72 h, with shaking (300 rpm) (Figure 4a). Following this, we characterized our samples using similar immunochemical methods as before (Figure 1). Our aim was to discern if cross‐seeded WT tau oligomers differ in their immunochemical properties from the unseeded ones. First, we performed dot‐blot analysis on our samples using the same four antibodies we previously used (Tau5, T22, TTCM‐1, TTCM‐2) (Figure 4b). Our results show that none of the cross‐seeded oligomer samples had differences in their affinity for the conformation‐specific antibodies: T22, TTCM‐1, or TTCM‐2 (Figure 4b). Additionally, this result was similar to that obtained with unseeded WT tau oligomer. We also subjected these samples to western‐blot analysis using Tau5 and T22 antibody to ascertain the different species present within them (Figure 4c,d). With Tau5, an almost identical banding pattern is seen in all the samples (Figure 4c). This suggests that the addition of mutant oligomers does not generate distinct aggregation species. However, upon probing our samples with T22 antibody, we see a prominent smearing pattern above 50 kDa only in the cross‐seeded samples compared to the unseeded one (Figure 4d).

### 2.5 Morphology of cross‐seeded WT tau oligomers is similar to unseeded ones but have distinct secondary structure

**FIGURE 5:**
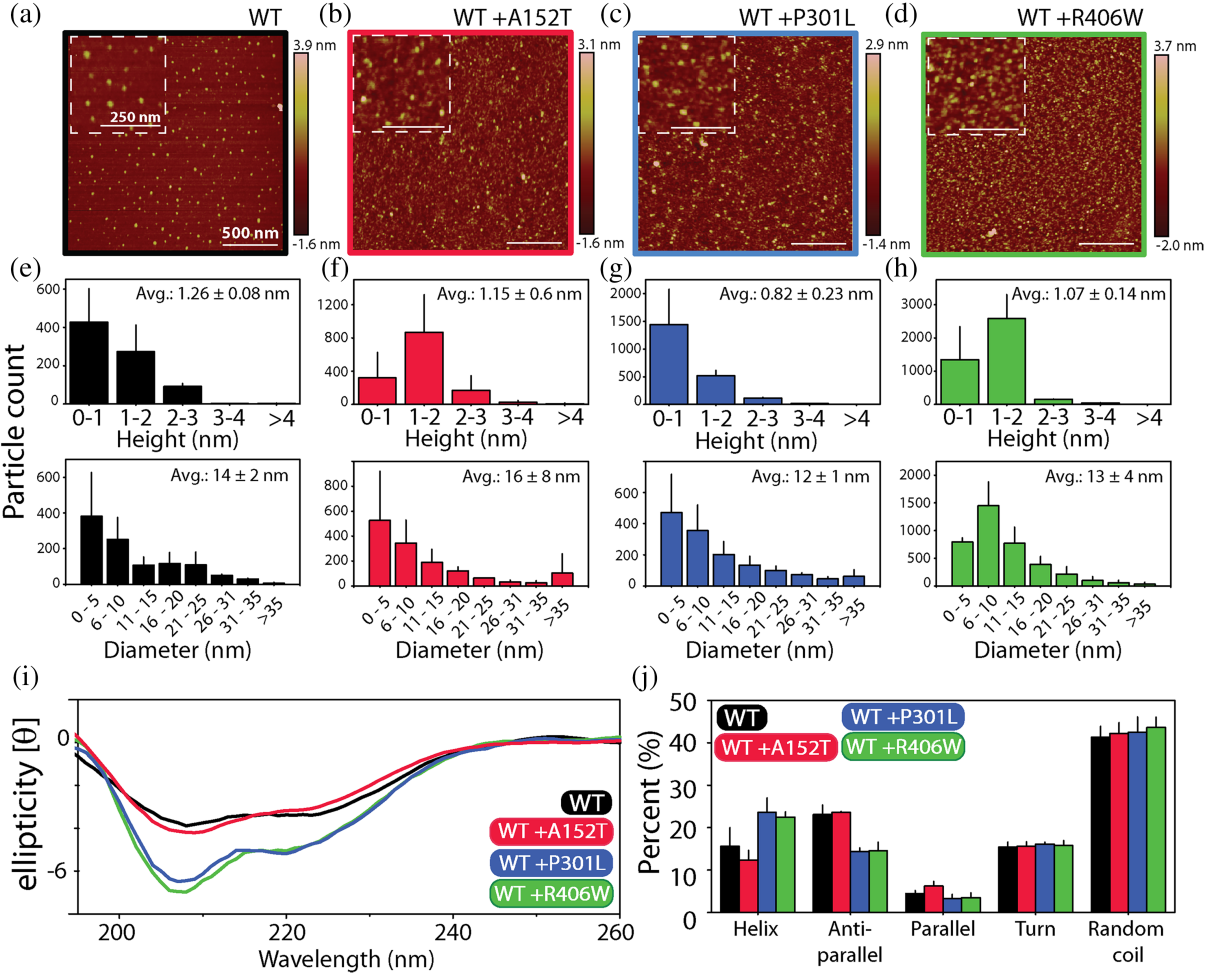
Morphology and secondary structure of cross‐seeded WT‐Tau oligomer is different from unseeded oligomers. (a–d) Atomic force microscopy analysis of oligomeric samples of unseeded WT tau (a, black), and cross‐seeded Tau: +A152T (b, red), +P301L (c, blue), and +R406W (d, green). Insets represent magnified areas on the field with the scale bar representing 250 nm, otherwise, the scale bar represents 500 nm in the full micrographs. (e–h) Dimensional parameters (height and diameter) of unseeded (e) and cross‐seeded WT tau oligomers (f–h) extracted using NanoScope v120rl software. (i) Far‐UV CD spectra of unseeded and cross‐seeded WT tau oligomers along with results from their deconvolution (j). Deconvolution was performed using the online BeStSel platform. Error bars represent a mean of triplicate readings. CD, circular dichroism.

Using AFM, we probed the morphology of the cross‐seeded WT tau oligomers and compared it to the unseeded sample (Figure 5a–h). The representative images of each sample are displayed in Figure 5a–d, and the particle analysis performed on these images are depicted in Figure 5e–h. As reported previously (Figure 2e), unseeded WT tau oligomers had dimensions of 1.26 ± 0.08 nm in height and 14 ± 2 nm in diameter (Figure 5e). The WT tau oligomers generated in presence of A152T oligomers had a height of 1.15 ± 0.6 nm and a diameter of 16 ± 8 nm (Figure 5f). In the presence of P301L oligomers, WT oligomers had a height of 0.82 ± 0.23 nm and a diameter of 12 ± 1 nm (Figure 5g), while in presence of R406W oligomers the WT oligomers attained a height of 1.07 ± 0.14 nm and a diameter of 13 ± 4 nm (Figure 5h). The margin of error in the readings tells us that there is no significant difference between the dimensions of cross‐seeded and unseeded WT tau oligomers, and they can be considered morphologically similar. We also analyzed and compared the secondary structure of the cross‐seeded and unseeded WT oligomers (Figure 5i,j). Visually, the far‐UV CD spectra of the WT oligomers cross‐seeded with A152T oligomers are similar to unseeded WT oligomers, while the WT oligomers cross‐seeded with P301L and R406W are similar to each other (Figure 5i). Upon deconvolution using BeStSel, we observe that random coils make‐up about 40%–50% of all the oligomers tested (Figure 5j). We see differences among the oligomers in the content of α‐helix and anti‐parallel β‐sheet: unseeded WT tau oligomers and WT tau oligomers cross‐seeded with A152T have a helical content of 15 ± 4% and 12 ± 2%, respectively, while those cross‐seeded with P301L and with R406W have a helical content of 23 ± 4% and 22 ± 1%, respectively (Figure 5j). The anti‐parallel β‐sheet content in unseeded WT tau oligomers and those cross‐seeded with A152T is 23 ± 2% and 23%, respectively, while in those cross‐seeded with P301L and with R406W oligomers is 14% and 14 ± 2%, respectively (Figure 5j). The parallel β‐sheet content in all the oligomers was fairly similar to each other, ranging from 3% to 6% (Figure 5j). Overall, the results confirms that there is similarity in secondary structures of unseeded WT tau oligomers and those generated in presence of A152T oligomers, and that they both differ from those generated in presence of P301L and R406W oligomers, which in turn are similar to each other.

### 2.6 Conformational differences exist among cross‐seeded and unseeded WT tau oligomers

**FIGURE 6:**
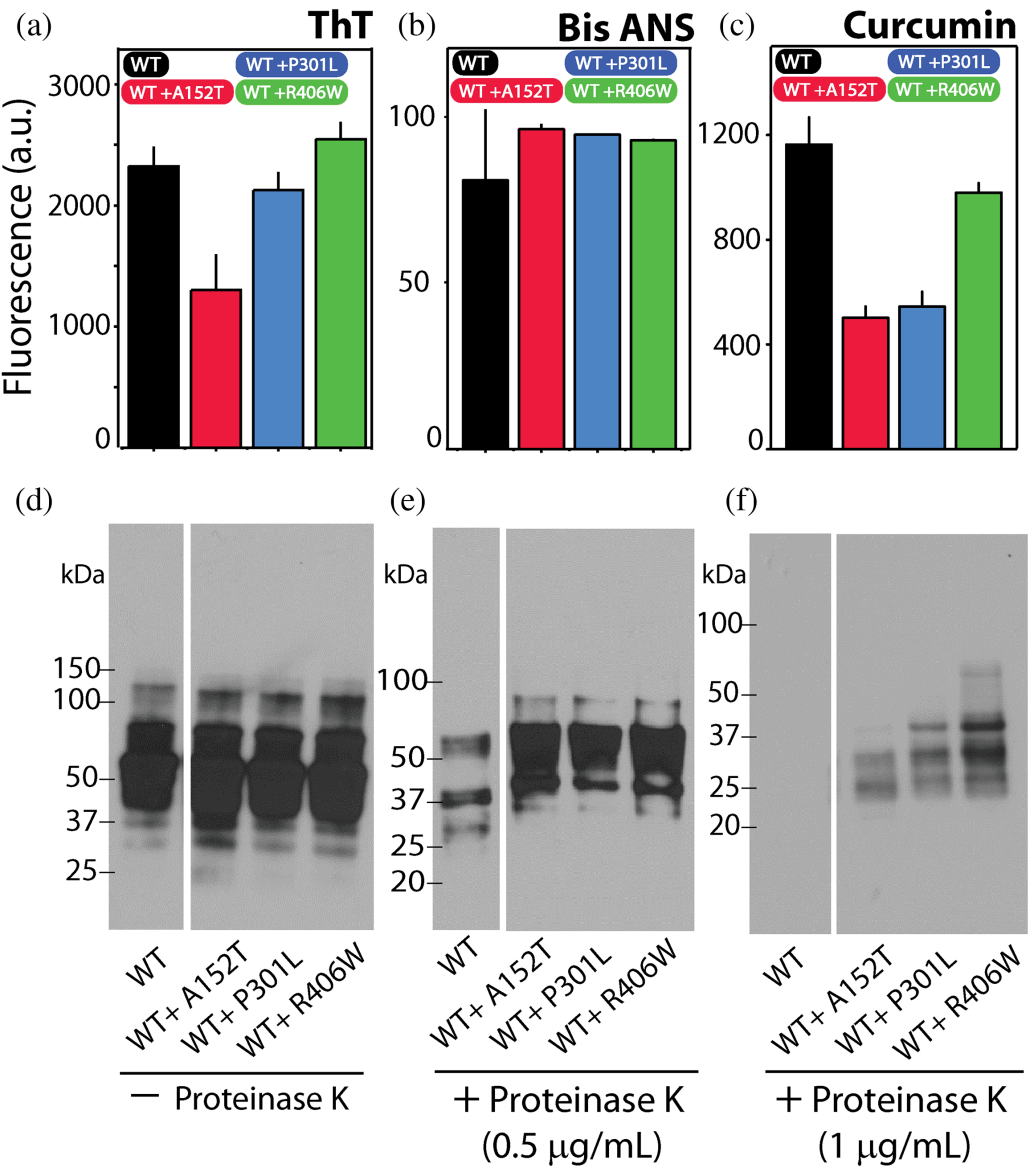
Cross‐seeded WT tau oligomers show conformational variation. (a–c) Fluorescence intensities of the dyes: ThT (a), bis‐ANS (b), and curcumin (c), in presence of cross‐seeded oligomers of WT tau. Error bars represent a mean of three repeats. (d–f) Proteolytic stability of cross‐seeded oligomers was tested by incubating them at 37°C for 30 min in presence of increasing concentrations of the enzyme proteinase K (PK): no PK (d), 0.5 μg/mL PK (e), and 1 μg/mL PK (f). The western blot analysis is consistent with three repeats. The lanes for WT tau alone (d and e) have been reproduced from Figure 3. Refer Figure S3 in Data S1 for complete, unedited blots.

To investigate potential conformational differences among the cross‐seeded and unseeded oligomers, we determined their dye‐binding properties and proteolytic stability (Figure 6). First, we measured the ThT fluorescence in presence of the oligomers to estimate their amyloid‐like content (Figure 6a). As reported previously (Figure 3a, WT tau), ThT fluorescence was 2323 ± 163 a.u. in presence of WT tau oligomers (Figure 6a). The fluorescence signal was lowest in presence of WT tau oligomers cross‐seeded with A152T oligomers; 1301 ± 297 a.u. (Figure 6a). In presence of oligomers cross‐seeded with P301L oligomers, the emission intensity was 2126 ± 151 a.u., and was 2548 ± 142 a.u. in presence of WT oligomers cross‐seeded with R406W oligomers (Figure 6a).

Following this, we measured the fluorescence intensity of bis‐ANS in presence of cross‐seeded WT tau oligomers (Figure 6b). Here, we saw noteworthy difference between the fluorescence intensity of unseeded WT tau oligomers compared to cross‐seeded ones; in the mixture with unseeded sample, bis‐ANS had an emission signal of 81 ± 21 a.u., while in sample cross‐seeded with A152T, it was 97 ± 1 a.u. In presence of oligomers cross‐seeded with P301L oligomers, the fluorescence intensity of bis‐ANS was 95 ± 0.3 a.u., and in presence of oligomers cross‐seeded with R406W oligomers, it was 93 ± 0.4 a.u. (Figure 6b). All the samples with cross‐seeded oligomers had higher intensity of bis‐ANS compared to unseeded sample.

Finally, we measured the fluorescence intensity of curcumin dye to complement preceding results (Figure 6c). As seen previously, in presence of unseeded WT tau oligomers, curcumin had a fluorescence intensity of 1164 ± 103 a.u. (Figure 6c). On the other hand, emission intensity of curcumin in presence of oligomer cross‐seeded with A152T oligomers was 501 ± 44 a.u., that with oligomers cross‐seeded with P301L oligomers was 545 ± 56 a.u., and finally, that in presence of oligomers cross‐seeded with R406W oligomers is 980 ± 37 a.u (Figure 6c). The results show that curcumin fluorescence intensity in presence of all the cross‐seeded WT oligomer samples was lower compared to unseeded WT tau oligomer sample.

We also measured the proteolytic stability of the cross‐seeded samples and compared it to unseeded WT tau oligomers samples that we discussed in Section 2.3 (Figure 6d–f). We used a similar experimental scheme as before (Figure 3d–f). Results show the intact oligomers of WT tau cross‐seeded samples with mutant oligomers in absence of PK (Figure 6d). We see prominent bands above and below 50 kDa, with a very similar pattern present in all samples (Figure 6d). Upon incubation with 0.5 μg/mL PK, we see digestion of oligomers in all samples, with it being less so in the cross‐seeded samples (Figure 6e). A faint band close to the 100 kDa mark is visible only in the cross‐seeded samples and is not seen in the unseeded sample (Figure 6e). In presence of 1.0 μg/mL PK, we see differences in the proteolytic stability of all the samples (Figure 6f). The sample of unseeded WT tau oligomer is completely digested, with no visible bands. In the WT tau oligomer sample cross‐seeded with A152T oligomer, bands are observed between 25 and 37 kDa, while in those cross‐seeded with P301L oligomers, we see similar bands but with differing intensities, along with a prominent band just above 37 kDa (Figure 6f). Finally, in sample of WT tau oligomer cross‐seeded with R406W oligomer, we see very similar banding pattern as the sample cross‐seeded with P301L oligomer, but here, the intensity of the bands is much more prominent. Additional faint smearing pattern of bands is visible above 37 kDa, indicating the presence of higher molecular weight species that are PK resistant (at 1.0 μg/mL) (Figure 6f). The differences in the proteolytic stability of WT tau oligomers that were generated by cross‐seeding with mutant oligomers are evidence of conformational heterogeneity.

## 3 DISCUSSION AND CONCLUSIONS

In this study, we sought to identify the conformational variety within WT tau oligomers, mutant tau oligomers, and the potential cross‐seeding among them. We focused our experiments on identifying structural differences in the oligomers rather than kinetic ones. Our studies utilized tau protein produced by bacterial systems and eschewed the use of heparin, in favor of generating oligomers in absence of external additives. For characterization, we used conformation‐specific tau oligomer antibodies, T22, TTCM‐1, TTCM‐2, that we previously generated within our laboratory (Lasagna‐Reeves et al., 2012; Montalbano et al., 2023). We see that all the oligomers we tested: unseeded, mutants, and cross‐seeded, have similar affinity for these antibodies (Figures 1 and 4). This result suggests that within these oligomers, the peptide backbone of the misfolded protein assumes a common, overall fold which is recognized by the conformation specific antibodies. Such a result is not unsurprising when one considers that the oligomer conformation specific antibody, A11, initially developed to specifically recognize Aβ oligomers, was shown to also recognize the oligomers of multiple other amyloid proteins (Kayed et al., 2003). We propose that oligomers we generated here provide a similar epitope for antibody binding, although differences exist elsewhere. Considering our most promising therapeutic strategies against neurodegenerative disorders are based on immunotherapy (van Dyck et al., 2022; Sevigny et al., 2016), results from such proof‐of‐concept studies add to our compendium on immunoreactivity of different amyloid aggregates.

Available literature is replete with examples of point mutants of amyloidogenic proteins which have unique structural and functional properties (Abedini et al., 2007; Flagmeier et al., 2016; Gendron et al., 2013; Hatami et al., 2017; Johnson et al., 2009; S. Lee et al., 2010; Pifer et al., 2011; Wolfe, 2009). Our AFM results parallel these findings by showing that oligomers of tau mutants have distinct morphological features compared to the WT tau oligomers (Figure 2). Although the differences were not significant, we observed that oligomers of tau P301L had the largest dimensions, while of those of A152T were the smallest among all the tau mutants. One limitation of our AFM results is that our measurements were made in air, which may not show the exact sizes of the oligomers and represents a distinct environment than the one in which they were generated (Mrdenovic et al., 2019; Ruggeri et al., 2019). However, our aim here is to understand the implication of mutations on the overall morphological differences of tau oligomers. In this context, further investigation is needed to understand the heterogeneity, stability, and dynamics of the oligomers in solution. The morphological differences we observe here might be a manifestation of the variability in secondary structure of the oligomers which potentially engenders an overall fold that is evident in AFM (Figure 2). We see noteworthy differences in content of α‐helix and anti‐parallel β‐sheet elements among these oligomers. Both of these structural elements are commonly adopted by soluble oligomers of other proteins as well (Bartels et al., 2011; Cerf et al., 2009; Tew et al., 2008; Zacco et al., 2018; Zou et al., 2013), and both are purportedly responsible for inducing toxicity by disrupting cellular membranes (Cerf et al., 2009; Ciudad et al., 2020; Pannuzzo et al., 2013). These differences aside, the BeStSel analysis shows that most of the oligomer retains the disordered, random coil dominated make‐up of the monomeric form (Figure 2). The persistence of dynamic flexibility in amyloid oligomers is an interesting feature since studies have correlated this to potential conformational alterations en‐route to fibrillation (Bhopatkar and Kayed, 2023; Cawood et al., 2021; Dear et al., 2020; Michaels et al., 2020; Morel et al., 2018). The flexibility afforded by a disordered chain means multiple, energetically equivalent conformations can sampled by the assemblies. Our oligomers retaining their random‐coil content potentially suggests that their final fibrillar structure is much more pliable and sensitive to its surroundings, compared to oligomers which are high in β‐sheet content. Additionally, this could also mean that the oligomers we visualize here are nascent forms, and that mature aggregates might be structurally rigid. Our eventual goal remains to characterize the higher molecular weight aggregates and fibrils that emerge from these oligomers; how conformationally different they are from each other, and to test how different factors such as pH, salt, osmolytes can affect the final form (Arar et al., 2024).

The use of fluorescent probes provides a convenient and sensitive tool to gather information on the conformational features of proteins (Hawe et al., 2008; Markus, 1965), and specifically, amyloids (Buell et al., 2010; Jameson et al., 2012). The shift in wavelength maxima (*λ*_max_) and to some extent, emission intensities, are indicators of the microenvironments and intrinsic fluorophores (polar, nonpolar) that are encountered by the fluorescent dye, which in turn indicates subtle differences in protein tertiary structure. In our results, we saw noteworthy differences in the fluorescent emission of all the dyes we tested against our monomeric and oligomeric samples (Figure 3). We used the ThT fluorescence assay to estimate the content of cross‐β‐sheet rich aggregates in our sample and further complement far‐UV CD analysis of the oligomers. Our results show that R406W oligomers had the highest ThT emission intensity among all mutant and WT tau oligomers while the WT tau oligomer sample had the lowest. In addition, *λ*_max_ and the emission intensities of bis‐ANS and curcumin also vary in presence of individual oligomers, further reiterating the presence of subtle conformational differences (Figure 3). However, one must keep in mind that there is a complex relationship between the interaction of dyes with amyloid structures and their fluorescent emission. For our case, such an emission is a response obtained in the presence of our oligomers (WT and mutants), which themselves have variable concentrations in solution and structural properties. As such, these results need to be considered holistically, in relation to other assays, to tease out the conformational properties. Therefore, we further complemented these studies with a proteolytic assay (Figure 3) (Li et al., 2021; Markus, 1965; Schönfelder et al., 2021). Bona fide amyloid fibrils, whether formed in vivo or in vitro, possess an amyloid core that is resistant to the action of proteolytic enzymes. Additionally, this assay provides a sensitive probe to discern conformational features in oligomers (Abd‐Elhadi et al., 2015; Dos Reis et al., 2002; Falcon et al., 2015; Lo Cascio et al., 2020; Novak et al., 1991; Saha et al., 2021). Our analysis showed striking differences in the proteolytic stability of all the oligomers we tested (Figure 3). In the presence of higher concentrations of PK, we saw the presence of proteolysis resistant species in oligomers of P301L, and which are less evident in those of A152T and R406W. Our results reveal that compared to the mutant tau oligomers, the WT tau oligomers are much more sensitive to degradation by PK.

Next, we investigated the ability of mutant oligomers to induce unique conformation in WT tau aggregates by their presence (Figures 4, 5, 6). The process of amyloid seeding involves addition/presence of pre‐formed amyloid aggregates (oligomers, protofibrils, or fibrils) to provide a template for aggregation/misfolding of monomeric units (Chaudhuri et al., 2019; Ivanova et al., 2021). For seeding purposes, we chose to maintain a seed/monomer ratio of 1/100 so as to minimize the effects of the seed. Except for immunochemical affinity and morphology (Figures 4 and 5), we saw differences in the secondary structure (Figure 5), dye‐binding ability (Figure 6), and proteolytic stability (Figure 6) of WT tau oligomers generated by cross‐seeding with oligomers of different mutants. Results from proteolytic stability are very striking in highlighting the conformational distinctness of the samples, revealing how unique PK resistant species are generated in the WT tau oligomers in the presence of each mutant oligomer (Figure 6). Overall, it is interesting to note that the cross‐seeded oligomers of WT tau did not propagate the conformational properties of the seed. Here, we can borrow the reasoning of Hartman et al. (2013) to explain this; the exact propagation of conformation between two different proteins is severely constrained by energetic parameters, where very strict compatibility between interacting sequences would be required. Our model, therefore, in a similar manner to theirs, might follow a non‐epitaxial heteronucleation, where ephemeral interactions between the foreign seed and monomer induces formation of a unique conformation but does not necessarily propagate its own (Hartman et al., 2013; Ivanova et al., 2021). Since our seed/monomer ratio is so low, we cannot speculate on whether the mutant seed integrates into the aggregates of the WT monomer (Petkova et al., 2005; Tycko, 2015). This report supports previous findings on the interaction of native tau and its mutants (Falcon et al., 2015; Strang et al., 2018) while also providing a novel perspective by keeping their conformational consequences at its focus. We show that point mutations in WT tau 2N4R lead to the generation of oligomers with distinct structural features. Additionally, our results indicate that oligomers of these point mutants can cross‐seed the WT tau monomer and induce generation of oligomers that are conformationally disparate from the unseeded form. From these results, one can speculate that a potential interplay between WT tau and its mutants can be a source of novel aggregates in familial tauopathies. An important future aim for us is to investigate the reciprocal effect of WT tau oligomers on the aggregation of mutant monomers. We speculate such an interaction will lead to generation of oligomers with unique conformation as well. A detailed analysis of the kinetic parameters of oligomer formation is also underway and would complement the results here by providing information on the aggregation pathway utilized. Furthermore, we are currently investigating the biological consequences of oligomeric polymorphism. We are probing whether each individual oligomer possesses a unique cytotoxic profile, which would give credibility to the idea that polymorphism among amyloid species underlies phenotypic variations observed in neurodegenerative disorders (Patel et al., 2022; Tycko, 2015). This report represents an important first step in understanding the conformational variety that exists between WT tau and its mutants, A152T, P301L, and R406W, while also shedding light on their cross talk.

## 4 MATERIALS AND METHODS

### 4.1 Recombinant expression and purification of tau

To generate the mutants of tau 2N4R: Tau A152T, P301L, and R406W, we used the WT tau sequence in the pET29b vector as the template. Point mutations were introduced using site‐directed mutagenesis at the Florida State University cloning facility. Recombinant expression and purification of tau 2N4R was performed as previously described (Margittai and Langen, 2004). Briefly, we used *E. coli* BL21 (DE3) competent cells (New England Biolabs) to express desired protein using the introduced plasmid (pET29b). Cryopreserved, transformed *E. coli* were grown in a small volume (10–25 mL) of Luria‐Bertani (LB) broth, supplemented with 1 mg/mL kanamycin termed as the primary culture. The primary culture was allowed to grow for 16 h at RT, while shaking at 200 rpm. Following this, we transferred the primary culture to 1 L of LB broth, also supplemented with 1 mg/mL kanamycin. The cells were grown at 37°C with shaking at 225 rpm till the OD_600_ reached a value of 0.6–0.8, following which protein over‐expression was induced using 1 mM IPTG (isopropyl β‐d‐1‐thiogalactopyranoside) and incubating at RT, while shaking at 175 rpm for ~16 h. Cells were harvested by centrifugation at 10,000 rpm for 10 min at 4°C. The cell pellets were resuspended in 25 mL lysis buffer (20 mM Tris, 500 mM NaCl and pH 8.0) supplemented with one cOmplete™, EDTA‐free Protease Inhibitor Cocktail (Sigma‐Aldrich) and 150 mM PMSF. The cells were lysed by sonication; 45°s burst, followed by 1 min rest on ice, which was repeated 10 times. Following this, we centrifuged the lysate at 10,000 rpm for 10 min at 4°C. We collected the supernatant and boiled it at 90°C to precipitate contaminants. We centrifuged the boiled supernatant at 10,000 rpm for 10 min at 4°C. The supernatant was collected and incubated with HisPur™ Ni‐NTA Resin (Thermo Scientific) for a minimum of 30 min at 4°C. The mixture was then packed into a Kontes® flex‐column. The column was washed with 2 bed volumes of wash buffer (20 mM Tris, 500 mM NaCl, 10 mM imidazole and pH 8.0) and the protein was eluted in 1 bed volume of elution buffer (20 mM Tris, 500 mM NaCl, 150 mM imidazole and pH 8.0). Following this, we exchanged the elution buffer for 1× PBS, using Slide‐A‐Lyzer™ Dialysis Cassettes, 10 kDa MWCO (Thermo Scientific). We checked the purity of each prep using SDS‐PAGE followed by silver staining. The concentration of our protein was estimated using BCA assay and the protein was suitably aliquoted and snap‐frozen for storage at −20°C until further use.

### 4.2 Aggregation of tau and cross‐seeding reactions

For generation of Tau oligomers (WT and mutants), we incubated 20 μM monomer of each protein in 1× PBS (Gibco) at 37°C for 72 h while shaking the mixtures at 300 rpm. For these reactions, we used low‐retention micro‐centrifuge tubes (Fisher brand). The presence of oligomers within our samples was confirmed by western‐blot analysis with Tau5 antibody. For cross‐seeding reactions, we used a similar setup; 20 μM monomer of WT tau was incubated with 200 nM mutant oligomer at 37°C for 72 h, while shaking at 300 rpm. The presence of oligomers was again confirmed by western‐blot analysis with Tau5 antibody.

### 4.3 Immunochemistry

For samples to be resolved on the basis of molecular weight with minimum interference to conformational integrity, we used precast NuPAGE Bis‐tris gel (Invitrogen). Mini‐PROTEIN electrophoresis unit (Bio‐Rad) was used to run the gel. Following this, we used Bio‐Rad Criterion Western Blotter Transfer to transfer protein onto a 0.45 μm nitrocellulose membrane (Cytiva Amersham™ Protran™). The membrane was blocked with 10% nonfat milk in TBS with low tween (TBST) for 1.5 h at RT. After that, each membrane was probed with individual antibody (Tau5, T22, TTCM‐2, and TTCM‐2) diluted in 5% nonfat milk and incubated for 1 h at RT. The primary antibody was removed and membrane washed three times with TBST for 10 min and incubated for 1 h at RT with anti‐mouse secondary antibody diluted in TBST (1:20,000). ECL developer solution (Advansta WesternBright™ ECL) was applied for 2 min before signal detection on x‐ray film.

### 4.4 AFM

AFM images of oligomer of WT tau 2N4R and its mutant were obtained using a non‐contacting tapping method with Bruker Multimode 8 AFM. A 12 mm mica fixed on a 15 mm metallic disc was freshly cleaved to obtain a uniform surface; 10 μL of 1:10 dilution sample (0.03–0.05 μg/μL) in PBS was applied on mica and allowed to adsorb overnight (~16 h at RT). The mica grid was then repeatedly washed with molecular grade water and dried in the air. A Scanasyst‐air probe (tip radius: 2–12 nm) was used with a peak force of 0.1 V at a frequency of 0.997 Hz in non‐contacting tapping mode to scan. Images were taken of at least three (maximum of five) different fields on the mica surface. Finally, images were analyzed using NanoScope Analysis v1.20rl AFM data processing software and were subjected to thresholding limits assigned for each dimension (diameter and height) and the processed data was plotted using Origin 8.5 (Origin Lab) graphing tool. Of note, the NanoScope v1.20rl software does not account for the contribution from the width of the AFM tip (Ruggeri et al., 2019).

### 4.5 CD

Far UV‐CD spectra of oligomer of recombinant Tau 2N4R and its mutant were scanned using Jasco J‐815 CD‐spectropolarimeter equipped with Peltier type temperature controller. For this, we used 0.1 mg/mL sample in a quartz cuvette of 1 mm path length. The measurement parameters were set at scan rate of 20 nm/min and an interval of 2 nm to record data from 195 to 260 nm with an average of three iteration used for each sample. The CD data were extracted using Spectra Manager provided by the manufacturer (Jasco Inc.) and plotted using Origin Pro 8.5. Data were de‐convoluted using BeStSel algorithm (Micsonai et al., 2015) and analyzed for secondary structure content using K2D2 algorithm (Micsonai et al., 2015, 2022). The experiment was performed in triplicates and data was processed using Origin 8.5.

### 4.6 Dye‐binding assays

ThT, curcumin, and bis‐ANS assays were performed on a POLARstar Omega plate reader (BMG LabTechnology). Briefly, 50 μL of each sample was added to a clear bottom 96‐well plate at a final concentration of 2 μM for each protein with either 10 μM ThT, 40 μM bis‐ANS, or 5 μM curcumin dye. ThT emission spectra were measured at an emission wavelength of 482 nm upon excitation at 450 nm, bis‐ANS emission was collected at 355 nm, and emission intensity was measured at 480 nm and for curcumin, the sample was excited at 450 nm and emission was monitored at 520 nm. Background corrections were performed by subtraction of baseline fluorescence signal of the buffer from corresponding samples. Each sample was tested in triplicates and data was processed using Origin 8.5.

### 4.7 Proteolysis assay

PK digestion of WT Tau 2N4R and its mutants were permed using previously published protocol with slight modifications (Lo Cascio et al., 2020; Sengupta et al., 2020). Protein was diluted with 5 mM NaCl and 100 mM tris, pH 8.0 and treated with two different concentrations of PK (0.5 and 1.0 μg/μL) and one negative control (no PK added) and incubated at 37°C for 1 h. Proteolysis was terminated by transferring the samples to ice and adding 4× SDS‐PAGE loading dye at a 1:3 ratio followed by incubation at 95°C for 5 min. Following this, the samples were electrophoresed using the protocol described above.

## AUTHOR CONTRIBUTIONS

**Anukool A. Bhopatkar:** Conceptualization; investigation; methodology; formal analysis; supervision; writing – original draft; writing – review and editing; visualization. **Nemil Bhatt:** Methodology; formal analysis; supervision; writing – original draft; writing – review and editing. **Md Anzarul Haque:** Investigation; writing – original draft; writing – review and editing. **Rhea Xavier:** Investigation. **Leiana Fung:** Investigation. **Cynthia Jerez:** Investigation. **Rakez Kayed:** Conceptualization; methodology; project administration; supervision; funding acquisition; resources; writing – review and editing.

## FUNDING INFORMATION

This work was funded by the National Institutes of Health grants: AG054025, AG10448132, and AG072458, and the Mitchell Center for Neurodegenerative Diseases.

## CONFLICT OF INTEREST STATEMENT

The authors declare no conflicts of interest.

## Supporting information


**Data S1.** Supplementary figures.
