# Supplementary material for: MAPT mutations associated with familial tauopathies lead to formation of conformationally distinct oligomers that have cross‐seeding ability
Source: Protein Sci. 2024 Aug 15;33(9):e5099. doi: 10.1002/pro.5099 (PMC11325167; doi:10.1002/pro.5099)
Supplement: Supplementary file 1 — Data S1. Supplementary figures. [file PRO-33-e5099-s001.docx]

**SUPPLEMENTARY INFORMATION**

**MAPT mutations** **associated with familial Tauopathies lead to formation of conformationally distinct oligomers that have cross-seeding ability.**

Anukool A. Bhopatkar^1,2,3,#^, Nemil Bhatt^1,2^, Md Anzarul Haque^1,2^, Rhea Xavier^1,2^, Leiana Fung^1,2,4^, Cynthia Jerez^1,2^, Rakez Kayed^1,#^

^1^Mitchell Center for Neurodegenerative Diseases, University of Texas Medical Branch, Galveston, Texas, USA; ^2^Departments of Neurology, Neuroscience and Cell Biology, University of Texas Medical Branch, Galveston, Texas, USA

**Current address**

^3^Department of Pharmacology and Toxicology, University of Mississippi Medical Center, Jackson, Mississippi, USA.

^4^Neuroscience Graduate program, UT Southwestern Medical Center, Dallas, Texas, USA.

**FIGURE S1**


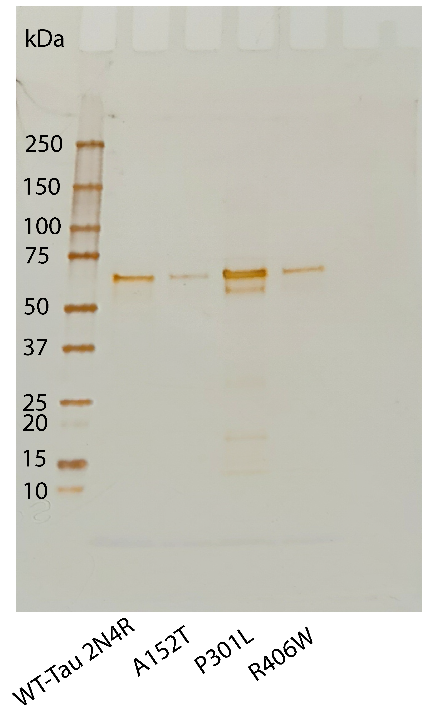

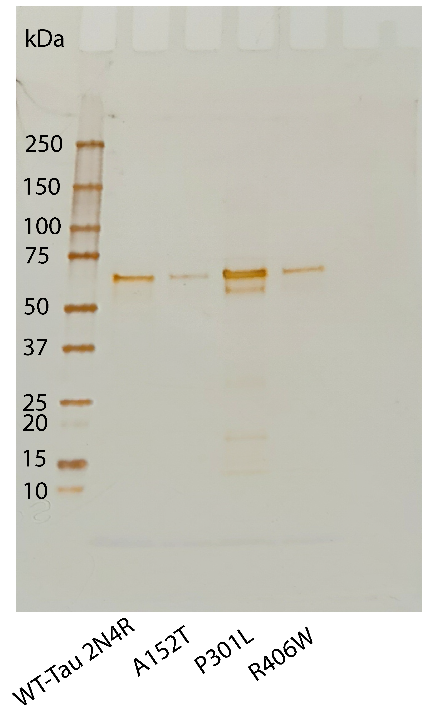


**FIGURE S1: Purification of recombinant Tau monomers:** SDS-PAGE used to characterize freshly purified monomers of WT-tau and mutants (A152T, P301L, and R406W). The protein samples were visualized using silver staining.

**FIGURE S2**


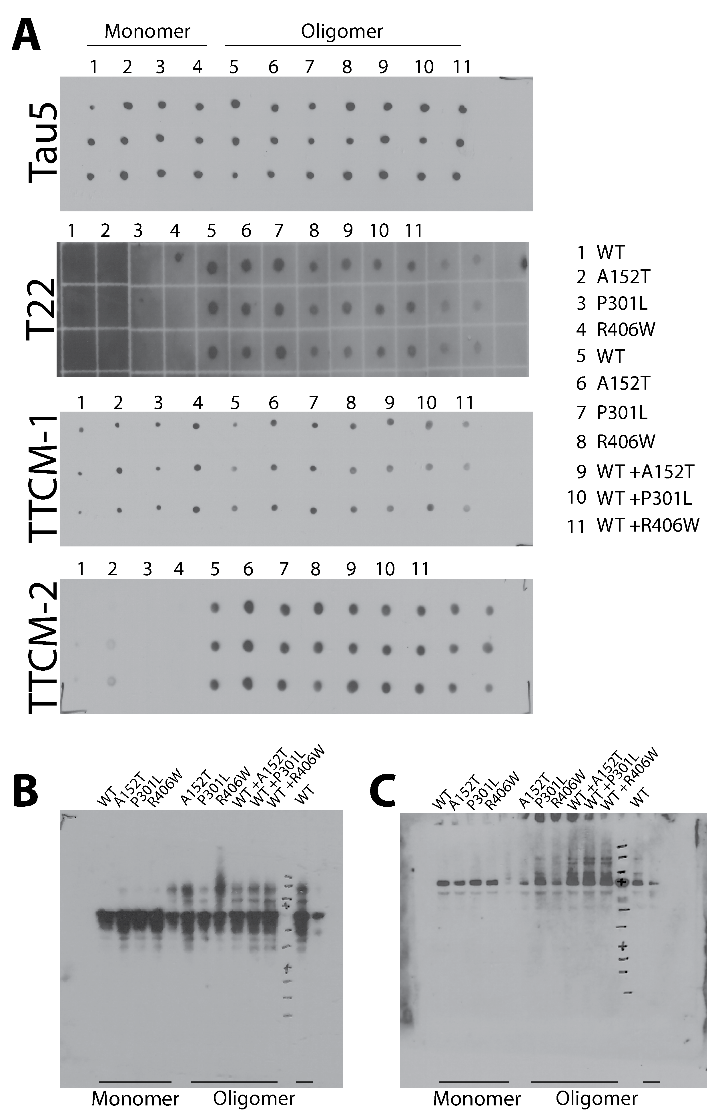


**FIGURE S2:** Immunochemical analysis of monomers and oligomers. A) Raw, unedited dot-blots probed with various antibodies. B-C) Raw unedited western-blots probed with Tau5 (B) and T22(C) antibodies.

**FIGURE S3**


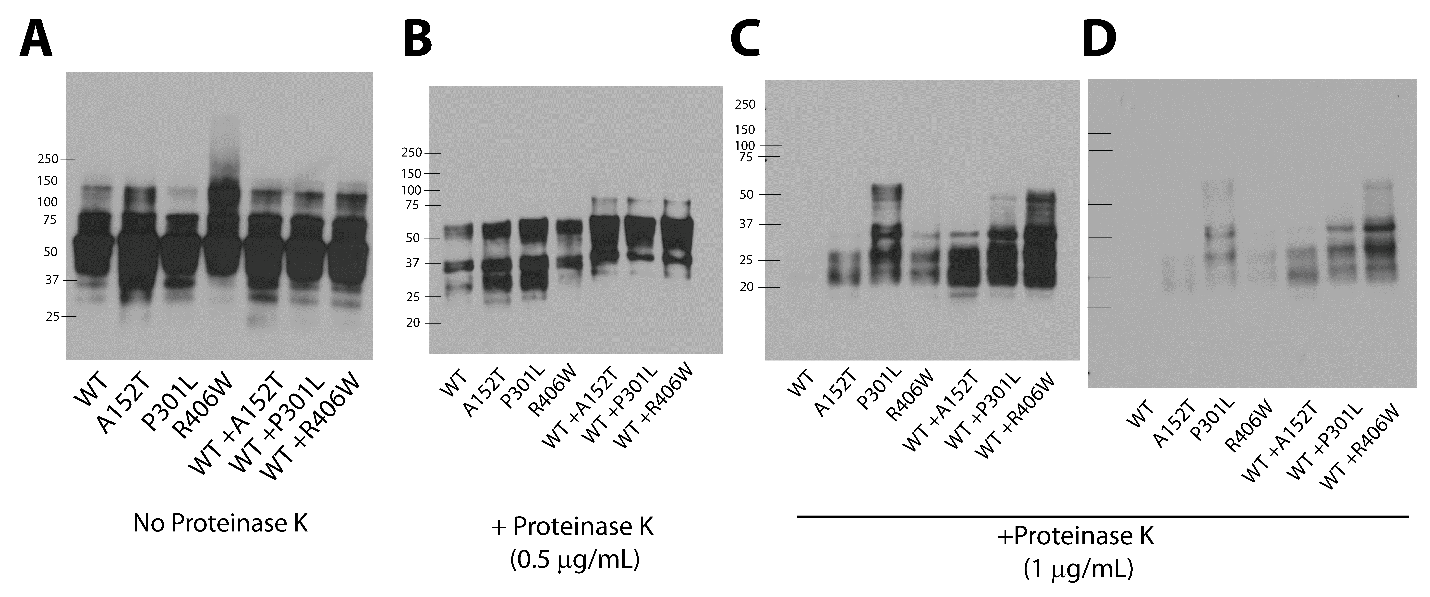


**FIGURE S3: Proteolytic stability of oligomers probed using proteinase-K.** A-D) Raw, unedited western blots for proteolytic stability of oligomers determined using varying concentrations of proteinase-K. The blots were probed using Tau5 antibody. C-D) Different exposures of the same blot to identify subtle differences between the banding patterns.

**FIGURE S4**


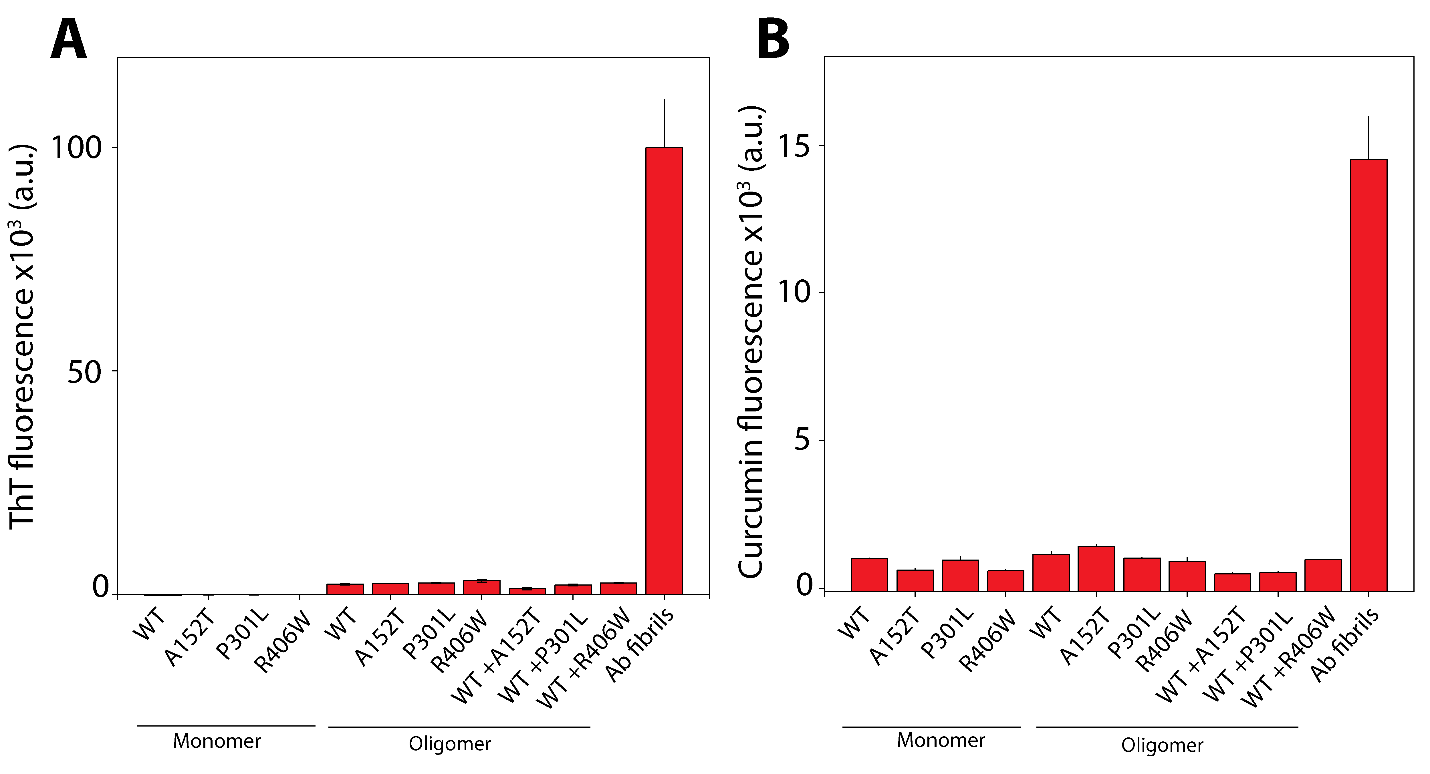


**FIGURE S4: Dye-binding ability of samples probed with ThT and Curcumin.** A-B) Magnitude of ThT(A) and curcumin(B) binding to different monomeric and oligomeric samples of Tau and its mutants. Included in these analyses is fluorescence intensities of the dyes in presence of amyloid-beta 42 (Aβ42) fibrils.
